# Integrated analysis of microRNA regulation and its interaction with mechanisms of epigenetic regulation in the etiology of systemic lupus erythematosus

**DOI:** 10.1371/journal.pone.0218116

**Published:** 2019-06-25

**Authors:** Elkin Navarro Quiroz, Roberto Navarro Quiroz, Lisandro Pacheco Lugo, Gustavo Aroca Martínez, Lorena Gómez Escorcia, Henry Gonzalez Torres, Andres Cadena Bonfanti, Maria del Carmen Marmolejo, Eduardo Sanchez, Jose Luis Villarreal Camacho, Hernan Lorenzi, Augusto Torres, Kelvin Fernando Navarro, Pablo Navarro Rodriguez, Joe Luis Villa, Cecilia Fernández-Ponce

**Affiliations:** 1 Universidad Simon Bolivar, Basic and Biomedical Faculty, Barranquilla, Colombia; 2 CMCC- Centro de Matemática, Computação e Cognição, Laboratório do Biologia Computacional e Bioinformática–LBCB, Universidade Federal do ABC, Sao Paulo, Brazil; 3 Clínica de la Costa, Department of Nephrology, Barranquilla, Colombia; 4 Universidad de Cadiz, Department of Biomedicine, Biotechnology and Public Health, Cadiz, Spain; 5 Universidad del Norte, School of Medicine, Barranquilla, Colombia; 6 Universidad Libre, School of Medicine, Barranquilla, Colombia; 7 Infectious Diseases Department, J. Craig Venter Institute, Rockville, Maryland, United States of America; 8 Universidad Popular del Cesar, Basic and Biomedical Faculty, Valledupar, Colombia; 9 ACCONP, Barranquilla, Colombia; 10 ColNalPinillos, Mompós, Colombia; 11 Procaps S.A., Research and Development, Barranquilla, Colombia; Peking University First Hospital, CHINA

## Abstract

The aim of this study was to identity *in silico* the relationships among microRNAs (miRNAs) and genes encoding transcription factors, ubiquitylation, DNA methylation, and histone modifications in systemic lupus erythematosus (SLE). To identify miRNA dysregulation in SLE, we used miR2Disease and PhenomiR for information about miRNAs exhibiting differential regulation in disease and other biological processes, and HMDD for information about experimentally supported human miRNA–disease association data from genetics, epigenetics, circulating miRNAs, and miRNA–target interactions. This information was incorporated into the miRNA analysis. High-throughput sequencing revealed circulating miRNAs associated with kidney damage in patients with SLE. As the main finding of our *in silico* analysis of miRNAs differentially expressed in SLE and their interactions with disease-susceptibility genes, post-translational modifications, and transcription factors; we highlight 226 miRNAs associated with genes and processes. Moreover, we highlight that alterations of miRNAs such as hsa-miR-30a-5p, hsa-miR-16-5p, hsa-miR-142-5p, and hsa-miR-324-3p are most commonly associated with post-translational modifications. In addition, altered miRNAs that are most frequently associated with susceptibility-related genes are hsa-miR-16-5p, hsa-miR-374a-5p, hsa-miR-34a-5p, hsa-miR-31-5p, and hsa-miR-1-3p.

## Introduction

Systemic lupus erythematosus (SLE) is an incurable systemic autoimmune disease that predominantly affects young women. Patients produce autoantibodies to double-stranded DNA (dsDNA), ribonucleoproteins (RNPs), cardiolipin, and phospholipids, among others, which form immune complexes (ICs) that are deposited in several different organs, such as skin, joints, and kidneys; leading to rashes, arthritis, and lupus nephritis (LN)[1][2].

Epigenetics is defined as heritable changes in gene expression without a change in the DNA sequence itself[3]. DNA methylation is a process typically used by mammalian cells to maintain a normal expression pattern; it is involved in the regulation of imprinted gene expression and X chromosome inactivation, among others[4]. DNA methylation almost exclusively occurs at the cytosine in CpG dinucleotides and is achieved by the addition of a methyl group to the 5′ position of a cytosine ring mediated by DNMTs. In mammalian genomes, around 80% of CpG cytosines are methylated, and they are asymmetrically distributed in the CpG poor and dense regions called CpG islands[5].

Associations have been revealed between abnormal epigenetic changes, such as DNA hypermethylation with human diseases and miRNAs regulated by more than one miRNA[6]. Recent evidence also suggests that they affect histone modifications. Maison *et al*. showed that RNase treatment can abolish the localization of methylated H3 lysine 9 and HP1 to pericentromeric chromatin[7]. Fukagawa *et al*. showed that Dicer-related RNAi machinery is necessary for the formation of heterochromatin structures, so miRNAs could also play important roles in controlling DNA methylation and histone modifications[8].

Recent data have demonstrated striking alterations of DNA methylation, histone modifications, and deregulated microRNA expression in patients with SLE, the sum of which contributes to the overexpression of selected autoimmune-related genes and a immunological self-tolerance loss[9][10]. Although previous studies showed that miRNAs can regulate DNA methylation by targeting the DNA methylation machinery, the role of miRNAs in aberrant CD4^+^ T-cell DNA hypomethylation in lupus remains unclear[11].

The current study aimed to identify *in silico* the likely interactions between miRNAs, genes encoding transcription factors, ubiquitylation, DNA methylation, and histone modifications in SLE.

## Materials and methods

### Identification of miRNAs potentially involved in the regulation of signaling pathways, in patients with SLE

To identify miRNA dysregulation in SLE, we used miR2Disease and PhenomiR to obtain information about miRNAs exhibiting differential regulation in diseases and other biological processes [12][13]. We also used the Human microRNA Disease Database (HMDD) in order to acquire information about experimentally supported human miRNA–disease association data from genetics, epigenetics, circulating miRNAs, and miRNA–target interactions[14]. We incorporated the obtained data into the analysis of miRNA results. High-throughput sequencing revealed circulating miRNAs associated with kidney damage in patients with SLE[15][16].

### MicroRNA target prediction and pathway analysis

For microRNA target prediction and pathway analysis, we used DIANA-miRPath (v3.0), a web-based application, to perform the enrichment analysis of genes predicted to be targeted by one or more miRNAs in biological pathways[17]. Two algorithms, namely, microT-CDS and miRTarBase, were used to predict miRNA targets. The software performs an enrichment analysis of multiple miRNA target genes to all known Kyoto Encyclopedia of Genes and Genomes (KEGG) pathways. The combinatorial effect of coexpressed miRNAs in the modulation of a given pathway was taken into account by the simultaneous analysis of multiple miRNAs. The graphical output of the program provided an overview of different parts of the pathway modulated by selected miRNAs, facilitating the interpretation and presentation of the analysis results. The statistical significance associated with the identified biological pathways was calculated using mirPath, which is available at http://microrna.gr/mirpath[18].

### Gene database construction enzymes that produce DNA and chromatin modifications

We used miRNet to establish genes associated with epigenetic modifications. miRNet offers a comprehensive tool suite to enable statistical analysis and functional interpretation of various data generated from current miRNA studies. miRNet is freely available at http://www.mirnet.ca.

**ENZYME** was also used to characterize enzymes that perform epigenetic modification functions. **ENZYME** was used with a repository of information related to the nomenclature of enzymes and is freely available at https://enzyme.expasy.org/[19]. To search the Enzyme Commission (EC) numbers and to identify the genes that belong to human, we used the gene-centered information from the NCBI sever[20].

### Database crush of microRNA target prediction and genes associated with epigenetic modifications

We used Access 2016 of Office package to cross the data obtained from the interrogation of the database of miRNAs target genes associated with SLE and the epigenetic regulation associated genes. The following matrix of data was obtained.

### Transcription factor target analysis

Validated TF–miRNA interactions and their regulation (activation or repression) were exported from the TransmiR database[21]. In contrast, predicted interactions of TF–miRNA and TF–gene were determined by retrieving the promoter sequences from all miRNAs and previously identified genes. The promoter region was defined as a 2-kb sequence starting 1.5 kb upstream from the transcription start site (TSS) and terminating 0.5 kb downstream of the TSS. TSS miRNA was obtained using miRStart[22].

### Regulatory network construction

In miRNA-based network, we included miRNAs, their targets, TFs regulating these miRNAs, and their targets, as well as, the type of interactions between these molecules. We assumed that all miRNAs repress their targets, unless otherwise indicated in TransmiR[21]. It is possible for miRNAs to activate their targets, but recent findings reveal that this is rare. We also assumed that TFs activate their targets, unless otherwise indicated in TransmiR. The gene-based network was created in a similar fashion. The networks were constructed and visualized using Cytoscape (version 3.2.0)[23].

## Results and discussion

### Human miRNA–SLE association dataset

**Fig 1:**
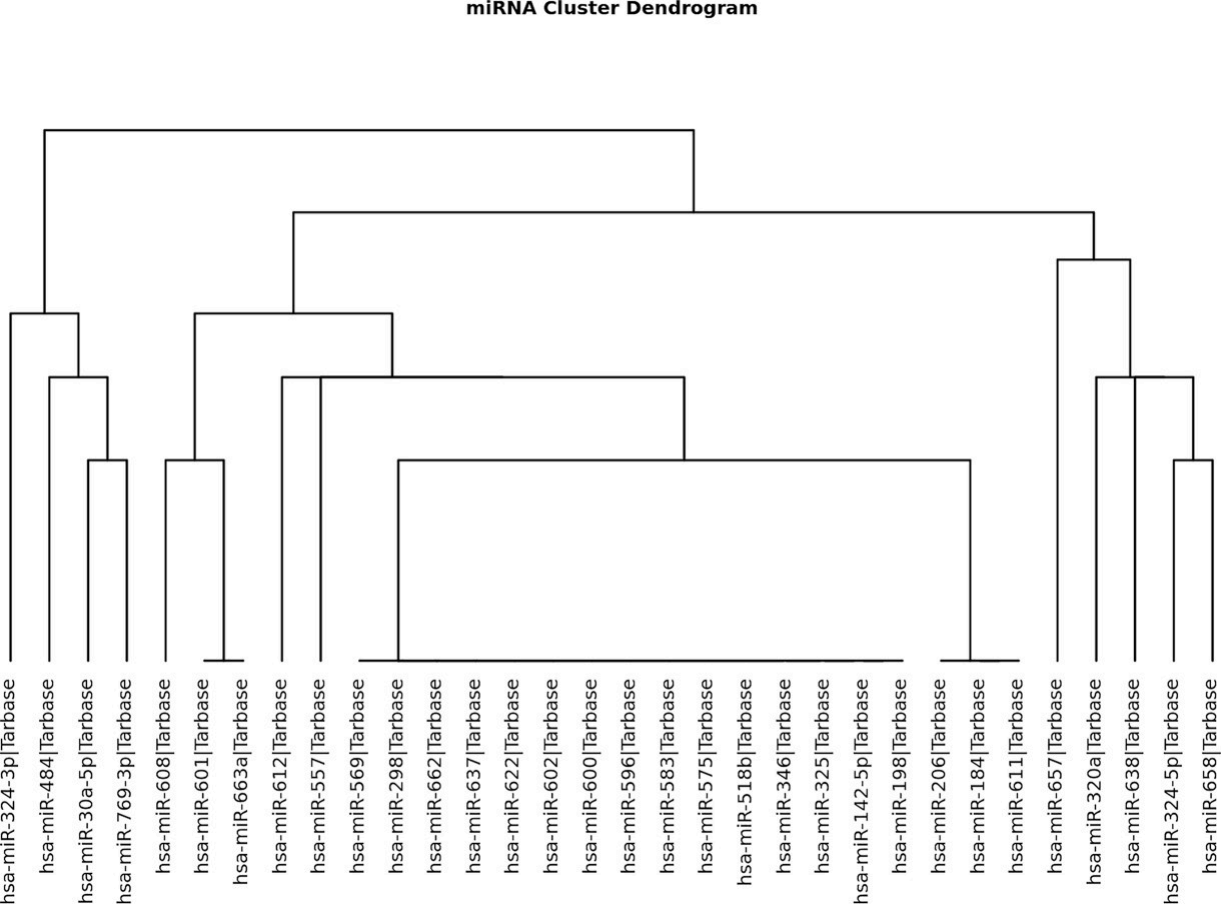
Cluster miRNAS: Dendrogram of results of cluster analysis of miRNAs related to SLE patients. This is the result from the interrogation of different databases in which the disease was associated with the regulation of these miRNAS, directly created from DIANA-miRPath v3.0 interface (http://www.microrna.gr/miRPathv3).

The miRNA–SLE association dataset was downloaded from HMDD v2.0[14], miR2Disease[12], and PhenomiR[13] databases. There were 95 miRNAs after filtering out duplicated records. We also incorporated data from a previous study, in which 21 Colombian individuals were enrolled [four patients with LN-IV, ten with SLE without nephritis (LNN group), and seven without autoimmune disease (including SLE) (controls)]. In this study miRNA profiles differences was analyzed using next-generation Illumina sequencing, and 69 differently expressed miRNAs were identified[15][16]. Moreover, 82 miRNAs were not included in the DIANA-miRPath v3.0 database[17]. Interestingly, the results data about miRNA cluster showed four clusters of miRNAs associated with each SLE risk factor, which means that four miRNA genes are located adjacent and theyare transcribed as one long pri-miRNA transcript and subsequently processed into the individual pre-miRNAs (Fig 1).

### Identification of target relationship miRNA–mRNA pairs

In this study, 5254 genes related to SLE susceptibility, and 141 miRNAs associated with SLE, as well as, their target genes from miRTarBase database were identified via experimental methods, such as luciferase or green fluorescent protein reporter assays, ELISA, qRT-PCR, and Western blot. Risk genes were taken from OMIM, GAD, and GWAS Central database. Target genes partially overlapped with risk genes, so Cytoscape software was used to visualize the relationship between susceptibility genes and SLE[23]. Parameters of SLE gene network were as follows: Clustering coefficient, 0.147; connected components, 465; network centralization, 0.134; average number of neighbors, 6,446; number of self-loops, 447; and shortest paths, 53%.

**Fig 2:**
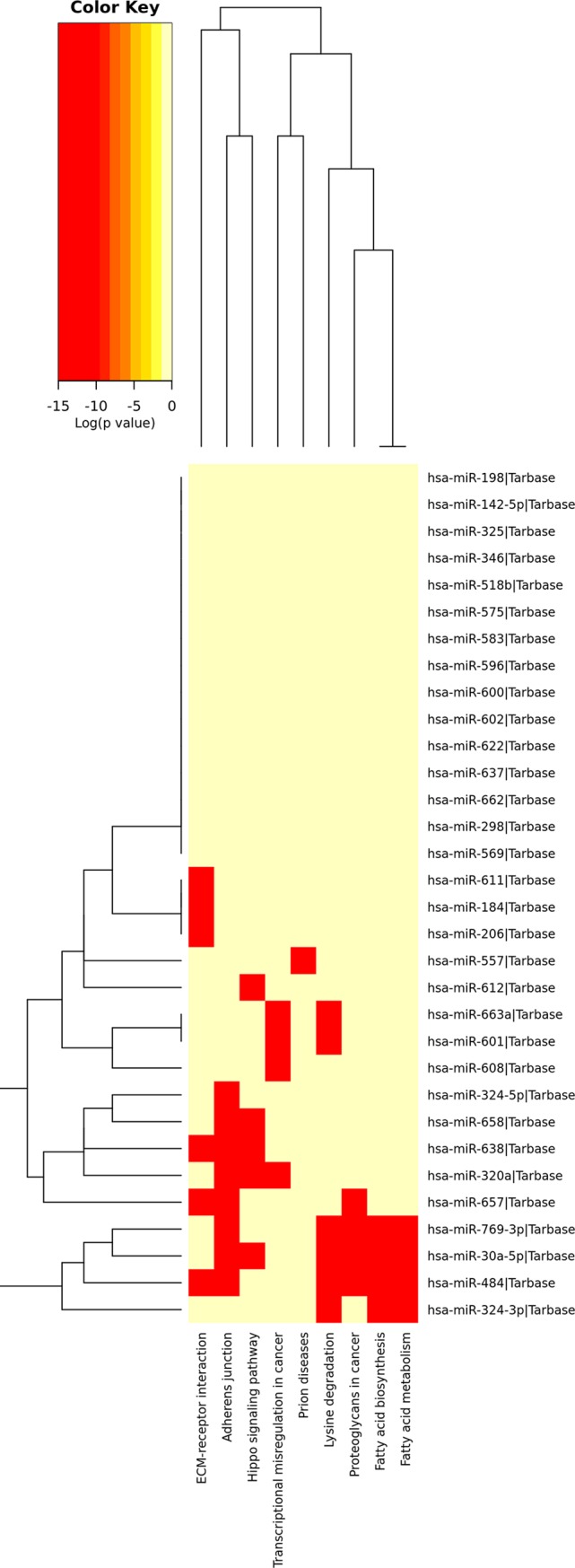
Heatmap of SLE: A miRNA versus GO Slim category heatmap *directly* created from DIANA-miRPath v3.0 interface. The heatmap depicts the level of enrichment of GO categories of several miRNAs in SLE patients, and enables the identification of miRNA subclasses or GO terms that characterize similar miRNAs because they are clustered together (http://www.microrna.gr/miRPathv3).

SLE risk pathways are associated with immune diseases or with the immune system, and highlight fundamental characteristics of autoimmune diseases. Using the cluster Profiler package, we identified 43 SLE-related pathways from KEGG database. The filter conditions were as follows: P-value < 0.01 and minimal size of genes annotated using ontology terms for testing >10. Based on the catalog of GO terms, the significantly enriched pathways included viral carcinogenesis (hsa05203), proteoglycans in cancer (hsa05205), pathways in cancer (hsa05200), TGF-β signaling pathway (hsa04350), ubiquitin-mediated proteolysis (hsa04120), p53 signaling pathway (hsa04115), Huntington’s disease (hsa05016), ECM–receptor interaction (hsa04512), and transcriptional misregulation in cancer (hsa05202) (Fig 2).

### Integrated mechanisms controlling the expression of proteins via post-transcriptional modification, which in turn are regulated by miRNAs, in SLE

**Fig 3:**
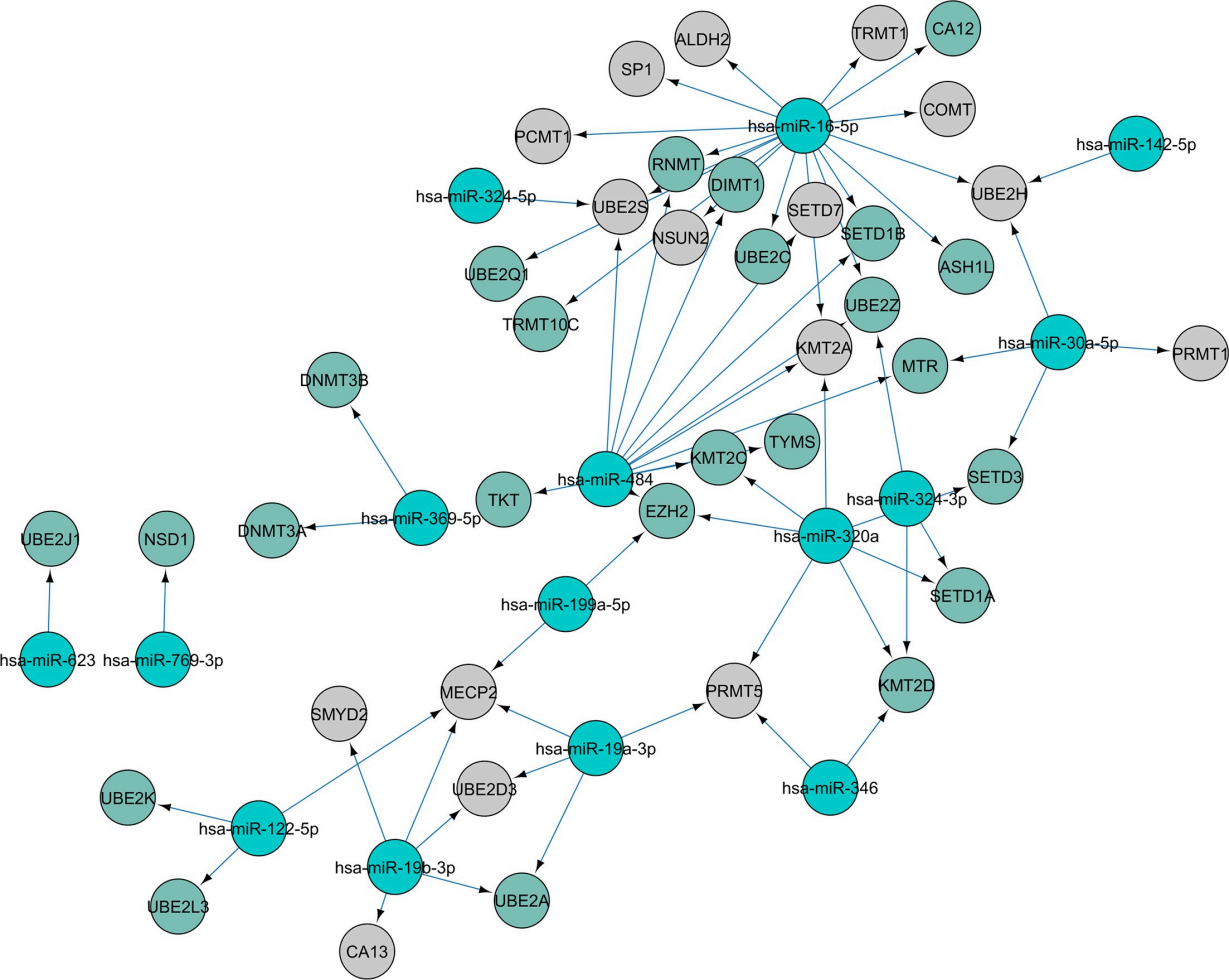
Networks of miRNAS with regular expression in patients with SLE and the associated proteins post-translational modifications (PTMs) coding genes. Green sphere corresponds to the name of the associated proteins post-translational modifications (PTMs) and gray spheres are miRNAS. On the other hand, arrows show regulation by miRNAS to the mRNA of associated proteins post-translational modifications (PTMs) coding genes.

Twenty-three miRNAs were identified to regulate the expression of 59 associated proteins post-translational modifications (PTMs), which are deregulated in patients with lupus erythematosus. In this work, we show networks of miRNAs and PTMs interactions resulting in 55 nodes, four connected components, 2% shortest paths, and 2,364 average number of neighbors (Fig 3).

It was estimated that 27.12% of PTMs were regulated by miRNAs that exhibited differential expression corresponding to the gene family ubiquitin-conjugating enzymes E2 (UBE2). Previous genome-wide association studies identified genetic variants in UBE2L3 region that are associated with SLE in subjects of European and Asian origin. UBE2L3 encodes an ubiquitin-conjugating enzyme, UBCH7, which is involved in cell proliferation and immune function. Significant associations were identified between variants in the region of UBE2L3 and SLE in subjects of European and Asian origin, which exceeded the Bonferroni-corrected threshold (P < 1 × 10^−4^)[24].

Ubiquitination is a critical post-translational protein modification for regulating NF-κB signaling[25]. However, little is known regarding the effect of UBCH7-mediated ubiquitination on NF-κB signaling. In a cell-free system, Orian *et al*. demonstrated that NF-κB precursor protein, p105, was a substrate for UBCH7-mediated ubiquitination. At rest, p105, encoded by the gene NF-κB1, undergoes constitutive proteasomal processing to yield the NF-κB subunit p50[26].

For genes related to methylation, 8.26% of PTMs were from the methyltransferase group (MECP2, PRMT5, DNMT3B, DNMT3A, TRMT13, TRMT1, TRMT10C, PCMT1, METTL3, MTR, DIMT1, PRMT1, METTL16, METTL15, NSUN2, Smyd2, TYMS, MECP2, and SETD3). In previous studies of global behavior of the deregulation of DNA methylation content, it was shown how this is affected in many types of cells, in a range of autoimmune disorders[27]. This phenomenon has been widely studied in SLE. Global changes of DNA methylation content can have different effects, including gene expression alteration, imprinting signature modification, and endoparasitic sequence reactivation, all of which contributes to the breakdown of immune tolerance checkpoints. CD4^+^ T cells in patients with SLE are characterized by a significant loss in the total content of 5-methylcytosine, which correlates with decreased levels of DNMTs. Interestingly, the symptomatology is directly associated with reduction in the level of this epigenetic mark[10].

According to SLE organ damage, Zhao et al. have shown a significant enrichment in the hypomethylated genes of SLE CD4+ T cells and they suggest that the increased expression of ITGAM, ITGB2 and ROCK1 genes, which encode for leukocyte integrin subunits and serine/treonin kinase, respectively; could be due to hypomethylation. Thus, hypomethylated genes related with leukocyte extravasation and recruitment into tissues, such as, skin or kidney, could induce local inflammatory damage in SLE patients[10][28].Additionally, Zhao et al. found hypomethylation of genes such as NDUFS5, SDHC and COX6A2, involved in “mitochondrial dysfunction” pathways, in SLE patients. Thus, in these patients, DNA hypomethylation could be correlated with an increasing of the disease activity and organ damage due to mitochondrial dysfunction, and subsequently autoimmunity triggering by oxidative modification of self-antigens[10][29].

Moreover, it has been described the hypomethylation and overexpression, in CD4+ T cells from SLE patients with only skin lesions, of NOD-like receptors (NLRs) genes such as NLRP2, which inhibit the NFĸB activation induced by several pro-inflammatory stimuli, probably participating in inflammatory response modulation. Thus, NLRP2 hypomethylation may contribute to the pathogenesis of skin lesions of SLE patients[10].

Other researchers have shown that interferon-related genes, such as IFIT1, IFIT3, IFI44L, TRIM22, and BST2 are overexpressed in activated T cells as compared to naïve T cells, due to a lower methylation level[30].These findings correlate with CD4+ T cell-specific hypomethylation described in SLE patients in both active and quiescent stages, suggesting that CD4+ T cell-specific hypomethylation is related with disease phenotypes[31].

The global decrease in DNA methylation is also supported by the generation of autoreactivity *in vitro* and lupus-like disease *in vivo*, as a consequence of treatment with hydralazine, a drug that decreases DNA methylation levels[32]. Conversely, peripheral blood mononuclear cells from psoriatic patients are characterized by an increase in DNA methylation accompanied by DNMT1 upregulation; the presence of these features is directly associated with psoriasis area and severity index scores[33].

Global alterations in the content of 5-methylcytosine suggest changes in repetitive elements because they are the major contributors of CpG dinucleotides, to the genome[34]. Despite this evidence, little is known about the specific repetitive elements that are affected in autoimmunity. Regarding SLE, the 18S and 28S regions of ribosomal RNA genes, that are represented in several hundred copies, have been demonstrated to be demethylated[35].

**Fig 4:**
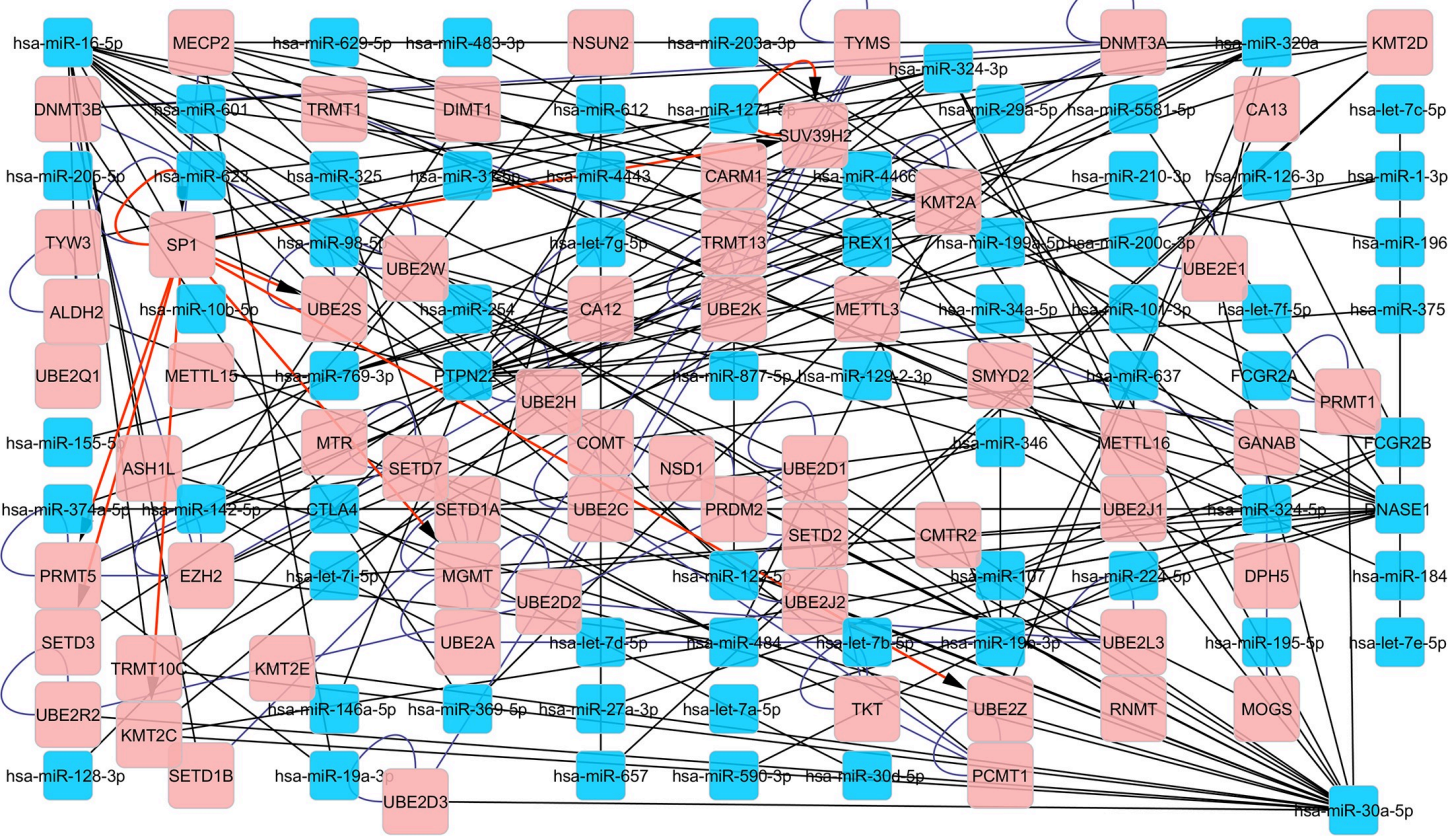
**Regulatory network in miRNAs of patients with SLE (Blue rectangles), associated SLE genes, and genes associated with post-translational modifications (Pink rectangles).** Black lines represent protein–miRNA interactions and red lines represent protein-protein interactions.

Another example of the contribution of PTMs to the etiology of SLE is glycosylation (TKT, GANAB, MBL2, and MOGS) (Figs 3 and 4). IKZF1, HLA-DQ2A/B, and BACH2 genetic loci that affect IgG glycome composition show a pleiotropic effect in SLE, indicating a potentially causative role of aberrant IgG glycosylation in SLE[36]. Furthermore, a decrease in IgG galactosylation has also been noted in several autoimmune and inflammatory diseases, where it probably plays a functional role and causes a decrease in the immunosuppressive potential of IgG. Fc galactosylation has recently been found to be necessary for the efficient association between FcγRIIB and dectin-1, leading to IgG anti-inflammatory activity[37]. Moreover, ICs rich in galactose residues have been found to inhibit the pro-inflammatory activity of the complement component C5a, which may represent another mechanism by which decreased IgG galactosylation participates in the pathology of autoimmune and inflammatory diseases[37].

Regarding miRNAs, miR30a-5p was frequently identified in the interrogated databases. Zhu *et al*. identified three commonly dysregulated miRNAs (miR-23b, miR-30a, and miR-214) in the affected tissues of patients with Rheumatoid arthritis (RA) or SLE, as well as in three mouse models of SLE, RA, and Multiple Sclerosis (MS), by comprehensively profiling miRNAs in local inflammatory lesions using human or mouse miRNA microarrays[38]. Similarly, in the present study, 22 genes regulating processes were identified, 40% were ubiquitin-protein ligases (PC00234) (e.g., UBE2H, UBE2D3, UBE2E1, UBE2D1, UBE2D2, UBE2H, UBE2D3, UBE2E1, UBE2D1, UBE2D2, UBE2R2, UBE2K, UBE2W, UBE2J2, UBE2R2, UBE2K, UBE2W, and UBE2J2), and 30% were DNA methyltransferases (PC00013) (e.g., CMTR2, NSD1, KMT2A, Suv39h2, and TRMT10C) (Fig 4).

In order to classify the diseases according to their different phenotypes or disease stages, MicroRNAs have emerged as biomarkers. It has been described the underexpression of miR-125a, in patients with SLE. MiR-125a is a negative regulator in the feedback loop of Kruppel-like factor 13 (KLF13) and RANTES production in activated T cells. Thus, in SLE patients, underexpression of miR-125a reduces RANTES levels, which has an essential role in lupus nepfropaty[39]. Moreover, other studies have shown the association of the differential expression of hsa-miR-371-5P, hsa-miR-423-5P, hsa-miR-638, hsamiR-1224-3P, hsa-miR-663, miR-26a and miR-30b, with lupus nephritis[40].Additionally, measuring miR-130b-3p level in serum from SLE patients has been demonstrated to be a diagnostic method to classify early and late stages of nephritis, and miR-150 has been shown to be a quantitative renal biomarker for kidney injury in SLE nephritis[41].

In a previous study, SUV39H2 and EZH2 mRNA were significantly downregulated in active lupus CD4^+^ T cells than in controls. Meanwhile, there was no significant difference in SUV39H1 mRNA levels between lupus CD4^+^ T cells and controls. A positive relationship between SUV39H2 mRNA and H3K9 methylation levels was also found. A recent research also identified that both H3K9 dimethylation and K27 trimethylation could be required for the establishment and maintenance of the inactive state of X chromosome[42]. Because one X chromosome is inactivated by processes, including DNA methylation, in women, the combined presence of H3K9 and H3K27 methylation may also be necessary to recruit additional activities, such as DNA methylation, which contribute to the stabilization of the inactive state[43].

Regarding SLE susceptibility genes, deoxyribonuclease I (DNase1) [36] has been shown to be regulated by 17 miRNAs (hsa-let-7a-5p, hsa-let-7e-5p, hsa-let-7i-5p, hsa-let-7c-5p, hsa-let-7f-5p, hsa-let-7g-5p, hsa-miR-98-5p, hsa-let-7b-5p, hsa-let-7d-5p, hsa-miR-374a-5p, hsa-miR-483-3p, hsa-miR-5581-5p, hsa-miR-629-5p, hsa-miR-122-5p, hsa-miR-107, hsa-miR-195-5p, and hsa-miR-203a-3p). DNase1 is a candidate endonuclease enzyme involved in nucleosome degradation in the apoptosis pathway, which suppresses anti-DNA autoimmunity. However, its function has been demonstrated to be diminished in patients with SLE[44][45].

Another gene associated with SLE susceptibility is three-prime repair exonuclease 1 (TREX1), which is regulated by six miRNAs (hsa-miR-1-3p, hsa-miR-34a-5p, hsa-miR-16-5p, hsa-miR-155-5p, hsa-miR-31-5p, and hsa-miR-374a-5p). hsa-miR-16-5p, hsa-miR-374a-5p, hsa-miR-34a-5p, hsa-miR-31-5p, and hsa-miR-1-3p regulate the gene DNase1 with TREX1. TREX1 is a widely expressed homo-dimeric protein, with no orthologs in lower eukaryotes, which preferentially degrades single-stranded DNA. Considering that recombinant TREX1 can act as a proofreading nuclease following oxidative DNA damage in vitro, the protein was proposed to play a role in DNA repair or replication. However, a subsequently derived Trex1-null murine model did not exhibit a predicted increase in cancer incidence, and rather than, these mice developed sterile inflammatory myocarditis, providing the first indication of a role of TREX1 in immune regulation[46].

## Conclusions

As the main finding of our *in silico* analysis of miRNAs with differential expression in SLE, they were shown to interact with susceptibility-related genes, post-translational modifications, and transcription factors. We highlighted 226 miRNAs associated with genes and processes, and alterations of miRNAs such as hsa-miR-30a-5p, hsa-miR-16-5p, hsa-miR-142-5p, and hsa-miR-324-3p which are frequently associated with proteins post-translational modifications (PTMs). Moreover, miRNAs most frequently associated with susceptibility-related genes were hsa-miR-16-5p, hsa-miR-374a-5p, hsa-miR-34a-5p, hsa-miR-31-5p, and hsa-miR-1-3p. This paper provides an overview of novel cellular and molecular mechanisms that seem to underlie the roles of miRNAs in SLE disease processes, clinical phenotype, as well as to indicate the future therapeutic potential of targeting miRNAs in the management of patients with SLE.

## Supporting information

S1 Table miRNAS associated with systemic lupus erythematosus found in HMDD. (XLSX)

S2 Table miRNAS associated with systemic lupus erythematosus found in miR2Disease. (XLSX)

S3 Table miRNAS associated with systemic lupus erythematosus found in PhenomiR. (XLSX)

S4 Table Circulating miRNAs whose abundance was significantly different between study groups. (DOCX)

S5 Table miRNAS associated with LES that have been associated proteins post-translational modifications. (XLSX)

S6 Table Post-translational modifications regulated by miRNAs. (XLSX)

S7 Table Proteins LES database DIANA-miRPath (v3.0) regulated by hsa-miR-142-5p. (XLSX)

## Abbreviations

DNase1: deoxyribonuclease I
dsDNA: double-stranded DNA
IC: immune complex
KEGG: Kyoto Encyclopedia of Genes and Genomes
LN: lupus nephritis; miRNA, microRNA
PTM: post-translational modification
RNP: ribonucleoprotein
SLE: systemic lupus erythematosus
TREX1: three-prime repair exonuclease 1
TSS: transcription start site
UBE2: ubiquitin-conjugating enzymes E2
